# Cost-effectiveness of adalimumab for early-stage Dupuytren’s disease: an economic evaluation based on a randomized controlled trial and individual-patient simulation model

**DOI:** 10.1302/2633-1462.311.BJO-2022-0103.R2

**Published:** 2022-11-16

**Authors:** Helen Dakin, Ines Rombach, Melina Dritsaki, Alastair Gray, Catherine Ball, Sarah E. Lamb, Jagdeep Nanchahal

**Affiliations:** 1 Health Economics Research Centre, University of Oxford, Oxford, UK; 2 Centre for Statistics in Medicine, Oxford Clinical Trials Research Unit, Nuffield Department of Orthopaedics, Rheumatology and Musculoskeletal Sciences, University of Oxford, Botnar Research Centre, Oxford, UK; 3 School of Health and Related Research (ScHARR), The University of Sheffield, Sheffield, UK; 4 Oxford Clinical Trials Research Unit, Nuffield Department of Orthopaedics, Rheumatology and Musculoskeletal Sciences, University of Oxford, Oxford, UK; 5 Department of Economics & Laboratory of Applied Economics, University of Western Macedonia, Kastoria, Greece; 6 Kennedy Institute, Nuffield Department of Orthopaedics, Rheumatology and Musculoskeletal Sciences, University of Oxford, Oxford, UK; 7 Faculty of Health and Life Sciences, University of Exeter, Exeter, UK

**Keywords:** Adalimumab, Anti-tumour necrosis factor, Cost-effectiveness analysis, Economic evaluation, Dupuytren’s disease, Palmar fibromatosis, Simulation model, Randomized controlled trial, randomized controlled trials, EQ-5D-5L, flexion deformities, radiotherapy, steroid injections, linear regression models, percutaneous needle fasciotomies, sensitivity analysis

## Abstract

**Aims:**

To estimate the potential cost-effectiveness of adalimumab compared with standard care alone for the treatment of early-stage Dupuytren’s disease (DD) and the value of further research from an NHS perspective.

**Methods:**

We used data from the Repurposing anti-TNF for Dupuytren’s disease (RIDD) randomized controlled trial of intranodular adalimumab injections in patients with early-stage progressive DD. RIDD found that intranodular adalimumab injections reduced nodule hardness and size in patients with early-stage DD, indicating the potential to control disease progression. A within-trial cost-utility analysis compared four adalimumab injections with no further treatment against standard care alone, taking a 12-month time horizon and using prospective data on EuroQol five-dimension five-level questionnaire (EQ-5D-5L) and resource use from the RIDD trial. We also developed a patient-level simulation model similar to a Markov model to extrapolate trial outcomes over a lifetime using data from the RIDD trial and a literature review. This also evaluated repeated courses of adalimumab each time the nodule reactivated (every three years) in patients who initially responded.

**Results:**

The within-trial economic evaluation found that adalimumab plus standard care cost £503,410 per quality-adjusted life year (QALY) gained versus standard care alone over a 12-month time horizon. The model-based extrapolation suggested that, over a lifetime, repeated courses of adalimumab could cost £14,593 (95% confidence interval £7,534 to £42,698) per QALY gained versus standard care alone. If the NHS was willing to pay £20,000/QALY gained, there is a 77% probability that adalimumab with retreatment is the best value for money.

**Conclusion:**

Repeated courses of adalimumab are likely to be a cost-effective treatment for progressive early-stage DD. The value of perfect parameter information that would eliminate all uncertainty around the parameters estimated in RIDD and the duration of quiescence was estimated to be £105 per patient or £272 million for all 2,584,411 prevalent cases in the UK.

Cite this article: *Bone Jt Open* 2022;3(11):898–906.

## Introduction

Dupuytren’s disease (DD) is a common fibrotic hand condition affecting 12% of 55-year-olds and 29% of 75-year-olds in Western populations.^1^ Early-stage DD presents as nodules on the palmar aspect of the hand that progress to form cords. The cords of late-stage DD cause curling of the finger joints (flexion deformity), impairing hand function and quality of life.^2^ Treatment options for late-stage DD include surgical excision, needle fasciotomy, and collagenase injections.^3^ However, all have limitations, including risk of recurrence.^4^ Although several interventions (e.g. steroid injections or radiotherapy) are used to treat early-stage DD, none have been compared against other interventions in randomized controlled trials (RCTs).^5^

We identified tumour necrosis factor (TNF) as a potential therapeutic target,^6,7^ and the RIDD phase 2a a dose-ranging trial found that injecting adalimumab (40 mg in 0.4 mL) downregulated myofibroblasts,^8^ the effector cells in DD. The RIDD phase 2b RCT found that adalimumab injections resulted in softening and reduction in size of early-stage progressive DD nodules, which continued to decrease further for nine months after the final injection.^9^ To date, RIDD is the only trial evaluating adalimumab in DD.

It is important to evaluate the extent to which the cost of early-stage DD treatment is offset by reductions in subsequent surgery and whether quality of life improvements are worth the additional cost. No previous studies have evaluated the cost-effectiveness of treatments for early-stage DD.^10^ Early economic evaluations are often used to identify whether treatments that are not yet licensed could be cost-effective and identify where future research would be most valuable.^11^

This study aimed to use RIDD trial data^12^ to assess the cost-effectiveness of adalimumab versus standard care in the UK NHS setting. The within-trial analysis compared costs and quality of life over one year. To determine longer-term outcomes, we built a patient-level simulation model to assess whether adalimumab (with/without retreatment) has the potential to be cost-effective for progressive early-stage DD, and estimate the value of further research.

## Methods

Full details of the RIDD (ISRCTN27786905, ClinicalTrials.gov NCT03180957) trial protocol^13^ and health economics analysis plan (HEAP)^12^ have been published. For both the within-trial and model-based economic evaluation, the patient population comprised adults with progressive early-stage DD who met the RIDD inclusion criteria: active extensor deficit ≤ 30° and an established, clinically distinct nodule with a clear history of progression in the preceding six months.^9^

Both analyses took an NHS and personal and social services (PSS) perspective, following the NICE reference case.^14^ Following UK guidelines,^14^ we conducted a cost-utility analysis, measuring health outcomes in quality-adjusted life years (QALYs). The economic evaluation compared adalimumab plus standard care against standard care alone, as most early-stage DD patients are currently not treated. The standard care group received placebo (saline) injection within the trial. The index year for costs was 2018 to 2019. Each course of adalimumab comprised four doses of 40 mg adalimumab in 0.4 ml (Humira; AbbVie, UK) administered at three-month intervals by a consultant in an outpatient setting, with the option of topical local anaesthetic cream. Each dose, including administration, cost £475 (Supplementary Table i).

### Within-trial economic evaluation

We assessed the cost-effectiveness of adalimumab versus standard care using QALYs as the main outcome, assessed by the EuroQol five-dimension five-level questionnaire (EQ-5D-5L) questionnaire^15^ at baseline, three, six, nine, 12, and 18 months. Resource use related to DD in the injected digit was assessed at the same timepoints, including injections and surgery performed and self-reported health and social care usage. An intention-to-treat cost-utility analysis was performed using data collected up to 12 months post-randomization; patients were analyzed on an as-randomized basis, regardless of adherence to the protocol. Missing data were handled using multiple imputation. Differences between treatment arms were estimated using linear regression models, adjusted for site, age, and (for QALYs only) baseline utilities. No discounting was used as the time horizon of primary interest was 12 months. We estimated the incremental cost-effectiveness ratio by dividing the mean cost difference between adalimumab and standard care by the mean QALY difference. The joint uncertainty around these estimates was explored using bootstrapping. We used sensitivity analyses to assess the impact of different drug costs, and adherence to the protocol. Linear regression and non-parametric bootstrapping were used to estimate model inputs from trial data. Supplementary Methods 1 provides additional details on unit costs and methodology for the within-trial analysis.

### Patient-level simulation model

**Fig. 1:**
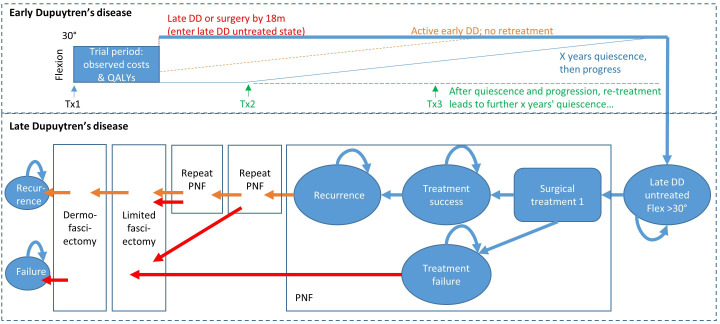
Model structure. The model simulates disease history for individual RIDD participants at discrete six-month time intervals. Once 18 months pass following initial treatment, individual patients either continue to have progressive early-stage Dupuytren’s disease (DD), enter quiescent early-stage DD, or progress to late-stage DD; the probability of each outcome varies between treatments. Patients with late-stage DD progress directly to the untreated late-stage DD state. Quiescent patients have no change in flexion deformity for a certain number of years (see Table I). After that period, they will either be retreated or have progressive early-stage DD (depending on treatment group). Patients with progressive early-stage DD experience progressive changes in flexion deformity (although these are shown as linear in the figure, in practice the change in flexion deformity during each six-month period varies stochastically and may be negative). When the flexion deformity exceeds 30°, patients enter the untreated late-stage DD state. In late-stage DD, patients may undergo a prespecified series of surgical procedures. In the base case analysis, this comprised up to three percutaneous needle fasciotomies (PNF), no more than one limited fasciectomy and no more than one dermofasciectomy. Patients who failed any PNF procedure and opted for further surgery received limited fasciectomy rather than PNF. Transitions in late-stage DD were modelled using the Markov disease states used by Brazzelli et al,^4^ although our model captures longer sequences of surgical interventions. The model also allows for death from any disease stage.

An individual-patient simulation model with a structure comparable to a Markov model was used to extrapolate individual-patient data from the RIDD trial. This provides early modelling of the lifetime cost-effectiveness of adalimumab and the value of further research. The structure of the model (Figure 1) was based on the conceptual model pre-specified within the HEAP,^12^ which was informed by a systematic review of previous economic evaluations.^10^ Table I and Supplementary Methods 2 give further details on the model, data inputs, and assumptions.

**Table I.** Key assumptions within the model. A full list of assumptions is given in Supplementary Methods 2.

Assumption
Six-month cycles.
55-year time horizon from randomization (equal to the life expectancy^16^ of the youngest UK patient randomized to placebo in RIDD).
Costs and utilities beyond the 18-month trial were discounted at 3.5% per annum.^14^
Late-stage DD was defined as flexion deformity > 30°. The transition to late-stage DD was assumed to be permanent and patients cannot return to early-stage disease.
Quiescent patients were assumed to have 0% chance of progressing to late-stage DD until quiescence ends. In the absence of external data on the duration of quiescence, the base case analysis assumed that quiescence lasts for double the duration of the trial (three years) regardless of whether quiescence was achieved with adalimumab treatment or without treatment. This was varied between the trial duration (1.5 years) and 5 years (approximately 3 times the trial duration) in one-way and probabilistic sensitivity analyses. The RIDD trial observed that adalimumab-treated nodules continued to soften and reduce in size over the nine-month period between end of treatment and 18 months,^9^ suggesting that the duration of quiescence is likely to be substantially longer than 1.5 years. Natural history studies have shown that many patients with untreated early-stage DD have periods of quiescence (during which the nodule is inactive and cords are unlikely to develop) and periods of activity (during which the nodule is more active and a cord may develop).^17^ The association between surface area and Tubiana stage (a categorical measure based on flexion deformity)^17^ supports the assumptions within the model regarding quiescence and the link between quiescence, the definition of which includes nodule size, and progression to flexion deformity.
As per the HEAP,^12^ we assumed that patients successfully treated for early-stage DD (defined here as quiescence) would not be at risk of disease progression for the duration of that quiescence.
Patients in the adalimumab retreatment group were assumed to seek treatment as soon as they noticed an increase in nodule size, pain, tenderness, or itching and those patients who were quiescent after the initial adalimumab treatment were assumed to maintain quiescence after retreatment; consequently, quiescence was assumed to continue indefinitely. Within RIDD, there was no relationship between the development of antibodies against adalimumab and response to treatment.^9^
Patients who did not reach quiescence with initial treatment were assumed to receive no further treatment for early-stage DD.
Patients who develop late-stage DD were assumed to receive up to three PNFs, no more than one limited fasciectomy and no more than one dermofasciectomy.^3^ PNF was only repeated if they relapsed after a PNF procedure that initially reduced flexion deformity to ≤5°; patients who failed any PNF procedure (postoperative flexion deformity >5°) and opted for further surgery received limited fasciectomy rather than PNF.
During the post-trial period modelled, no costs were applied for management of DD other than interventions (e.g. adalimumab, surgery and outpatient/physiotherapy consultations associated with surgery), since the costs were negligible within the trial.
The mean costs and QALYs accrued during the trial period were added to those estimated in the model to give lifetime costs and QALYs.
Patients with DD were assumed to have higher mortality than the general population based on a recent data linkage study, but DD stage and treatment were assumed to have no effect on mortality.^18^

DD, Dupuytren’s disease; HEAP, health economics analysis plan; PNF, percutaneous needle fasciotomy; QALY, quality-adjusted life year; RIDD, Repurposing anti-TNF for Dupuytren’s disease randomized controlled trial.

We extrapolated individual-patient data for 69 UK RIDD participants randomized to placebo with complete baseline data. The model simulated changes in flexion deformity and EuroQol five-dimension questionnaire (EQ-5D) utility of each individual over time, depending on treatments and disease progression. Each individual was simulated 100 times for each of 1,000 sets of parameter values under each treatment strategy to obtain stable estimates and minimize Monte Carlo error (Supplementary Methods 2). The model simulated disease progression and treatment of a single DD nodule from the end of the 18-month trial until death.

Nodule quiescence was used as an intermediate endpoint linking trial outcomes with progression to late-stage DD. Quiescence was defined as having all three of the following between baseline and 18 months: decrease or no change in nodule area; decrease or no change in nodule hardness; and ≤ 5° increase in active flexion deformity. Table I summarizes key model assumptions.

The model compared three treatment strategies: 1) standard care: no treatment for early-stage DD – a proportion of patients will be quiescent for three years, after which flexion deformity will gradually increase; 2) one course of adalimumab: patients have four adalimumab injections and receive no further treatment for early-stage DD – a proportion of patients will be quiescent for three years, after which flexion deformity will gradually increase; 3) repeated courses of adalimumab injections: patients who were quiescent after the initial four injections will maintain quiescence indefinitely by receiving further courses of four injections each time the nodule reactivates – patients who were not initially quiescent will receive no further treatment for early-stage DD.

RIDD data were used to calculate the probability of achieving quiescence or developing late-stage DD by 18 months, the effect of quiescence on quality of life, and the rate at which flexion deformity changes without treatment (Supplementary Methods 2). Data sources for model inputs that could not be reliably obtained from the RIDD sample were identified from a systematic review,^10^ supplemented, where necessary, by more recent data.^18-20^

Probabilistic sensitivity analysis quantified uncertainty, generating cost-effectiveness acceptability curves and estimates of the expected value of perfect information using published software^21^ (Supplementary Methods 2). All uncertain parameters were varied in one-way sensitivity analysis across their 95% confidence interval (CI) or the range reported in literature. Overall 13 sensitivity analyses were also conducted (Supplementary Methods 2). When assessing cost-effectiveness, we assumed that the NHS would be willing or able to pay up to £20,000 per QALY gained.^14^

There is no approved treatment for early-stage DD, although treatments such as intranodular steroid injections or radiotherapy are used despite the lack of comparative RCT evidence.^5^ The studies to date are poor quality, non-randomized, unblinded, and do not report objective measures of nodule size, hardness, or flexion deformity.^5^ The National Institute for Health and Care Excellence (NICE) found insufficient evidence to recommend radiotherapy outside research settings.^22^ Although there was insufficient evidence to robustly assess the cost-effectiveness of adalimumab compared with other active treatments, we conducted scenario analyses comparing with radiotherapy and steroid injections (Supplementary Methods 2). Collagenase has been evaluated in a small trial for early-stage DD,^23^ but was not considered here as it is only approved for late-stage disease and has previously been shown to not be cost-effective.^4,19^

Statistical analyses and the decision-analytical model were conducted in Stata release 17 (StataCorp, USA), except for simulating parameter values for probabilistic sensitivity analyses and taking percentiles across bootstraps, which were conducted in Microsoft Excel 2016 (Microsoft, USA).

## Results

### Within-trial economic evaluation

Two UK centres recruited 140 participants between February 2017 and April 2019.^9^ At 12 months, 128 (91%) participants had quality of life data and 130 (93%) had healthcare resource use (Supplementary Table ii).

EQ-5D-5L utilities remained relatively constant during the trial, with a mean between-group difference of 0.011 (95% CI -0.026 to 0.048) at 12 months (linear mixed model, Supplementary Table iii). Resource use among the trial population was low, with 2% (3/130) attending GP consultations, 2% (2/130) undergoing surgery, and no patients reporting NHS physio/hand therapy in months 9 to 12 (Supplementary Table v). Use of non-NHS resources was negligible (Supplementary Table x). Total QALYs and NHS/PSS costs other than adalimumab did not differ significantly between randomized groups over the 12-month follow-up (p ≥ 0.657, linear regression; Table II, Supplementary Table vii).

**Table II.** Results of the within-trial economic evaluation from baseline to 12 months

Outcome	Adalimumab, mean (SE)	Standard care, mean (SE)	Mean difference (95% CI)
n	70	70	N/A
QALYs *	0.875 (0.012)	0.855 (0.012)	0.004 (-0.019 to 0.027)
Total NHS and PSS costs baseline to 12 months (including intervention), £†	2,070 (53)	37 (27)	2,035 (1,919 to 2,152)
*Adalimumab injection costs, £* †	2,030 (43)	0 (0)	2,028 (1,944 to 2,112)
Incremental cost-effectiveness ratio (ICER): cost per QALY gained, £	N/A	N/A	503,410‡
Probability of cost-effectiveness at willingness to pay threshold of £20,000 per QALY (NHS and PSS perspective)‡	N/A	N/A	0%

The ICER was generated from un-rounded figures; therefore, the figure cannot be replicated exactly from the rounded figures shown in the table.

* Differences and p-values derived from linear regression model adjusted for age, site, and baseline utility score.

† Differences and p-values derived from linear regression model adjusted for age and site. The means for each group are unadjusted; and the difference between the unadjusted group means will therefore not equal the adjusted treatment effect.

‡ Adalimumab injections provide a small QALY benefit, but are more costly than standard care.

CI, confidence interval; N/A, not applicable; PSS, personal and social services; QALY, quality-adjusted life year; SE, standard error.

At a 12-month time horizon, adalimumab cost £503,410 per QALY gained and the probability of adalimumab injections being cost-effective at a willingness to pay threshold of £20,000/QALY was < 1% (Table II, Supplementary Figure a). Our sensitivity analyses confirmed the base case finding that adalimumab was unlikely to be cost-effective over a 12- to 18-month time horizon at any realistic cost for the adalimumab injections (Supplementary Tables viii to ix).

The analyses to estimate model inputs (Table III) showed that 21% of patients randomized to placebo had late-stage DD by 18 months and 22% were quiescent. Adalimumab-treated patients were 79% (95% CI -4% to 211%; p = 0.076, non-parametric bootstrapping) more likely to have quiescence at 18 months and marginally less likely to have late-stage DD or undergo surgery by 18 months (p = 0.534, non-parametric bootstrapping). Patients meeting the criteria for quiescence at 18 months accrued a mean 0.0395 (95% CI 0.0079 to 0.0704; p = 0.018, linear regression) more QALYs between six and 18 months than patients who did not have quiescence.

**Table III.** Model inputs estimated on RIDD data. Means and confidence intervals represent the mean, 2.5^th^, and 97.5^th^ percentiles across 1,000 bootstraps (estimated in Microsoft Excel 2016) and include multiple imputation of flexion deformity, nodule hardness, and nodule size. p-values represent the two-sided bootstrap p-value (2*(1-proportion of bootstraps < 0)). The methods of this analysis are described in Supplementary Methods 2, "Methods for the analysis of within-trial data used as inputs for the model" section.

Model parameter	Mean (95% CI)	p-value
Linear regression predicting QALYs between 6 and 18 months (all participants)		
Quiescence at 18 months	0.0395 (0.0079 to 0.0704)	0.018
Baseline EQ-5D utility	0.5410 (0.4015 to 0.6887)	
Constant	0.3847 (0.2581 to 0.5161)	
Linear regression predicting change in flexion deformity between baseline and 18 months (placebo group)		
Ectopic disease (plantar, Peyronie’s disease, or Garrod’s knuckle pads)	8.5189 (2.1683 to 15.3596)	0.006
Constant (Mean change in flexion deformity for patients without ectopic disease)	0.8631 (-2.0005 to 4.1074)	
Root-mean squared error	12.6079	
Mean change in flexion deformity for patients with ectopic disease (coefficient for ectopic disease, plus constant)	9.3821 (3.7979 to 15.3832)	
**Crude proportion of patients in each bootstrap with quiescence or late-stage DD (all participants)**		
Proportion of placebo patients with late-stage DD at 18 months*	0.2149 (0.1286 to 0.3143)	
Proportion of placebo patients quiescent at 18 months*	0.2209 (0.1286 to 0.3429)	
Proportion of adalimumab patients with late-stage DD at 18 months*	0.1800 (0.1000 to 0.2857)	
Proportion of adalimumab patients quiescent at 18 months*	0.3696 (0.2571 to 0.4857)	
Relative risk of quiescence with adalimumab vs placebo	1.7902 (0.9565 to 3.1111)	0.076
Relative risk of late-stage DD with adalimumab vs placebo	0.8881 (0.4000 to 1.6667)	0.534

*A total of 70 patients were randomized to each treatment group. Since multiple imputation was used to impute missing data on flexion deformity, nodule hardness, and nodule size, the number of patients meeting the criteria for quiescence or late-stage Dupuytren’s disease varied between imputed datasets and absolute numbers cannot be given for these proportions.

CI, confidence interval; DD, Dupuytren’s disease; EQ-5D, EuroQol five-dimension questionnaire; QALY, quality-adjusted life year.

### Patient-level simulation model

The simulation model suggested that over a lifetime, a single course of adalimumab delayed onset of late-stage DD by a mean 0.31 years (95% CI -0.51 to 1.31) compared with standard care. Further courses of four adalimumab injections each time quiescence ended (in patients who were initially quiescent) gained a mean 1.81 years (95% CI 0.51 to 3.10) free of late-stage DD by preventing disease progression in 37% of patients. Consequently, patients who received a single course of adalimumab required a mean 0.06 (95% CI -0.10 to 0.23) fewer operations for late-stage DD over a lifetime, compared with those who received standard care alone for early-stage DD (Table IV). Repeated adalimumab injections led to a mean 3.65 (95% CI 2.35 to 5.74) courses of four adalimumab injections and a mean 0.44 (95% CI 0.16 to 0.73) fewer operations for late-stage DD compared with standard care.

**Table IV.** Base case results of the model-based economic evaluation. Values represent means with 95% confidence intervals in brackets.

Model outcome	Standard care	1 course adalimumab	Repeated courses adalimumab
QALYs: Trial	1.279 (1.247 to 1.313)	1.286 (1.248 to 1.320)	1.286 (1.248 to 1.320)
QALYs: Model*	10.15 (9.80 to 10.49)	10.19 (9.86 to 10.51)	10.45 (10.10 to 10.79)
QALYs: Lifetime*	11.43 (11.08 to 11.78)	11.48 (11.14 to 11.80)	11.74 (11.40 to 12.08)
NHS costs, £: Trial	307 (134 to 514)	2,136 (1,998 to 2,277)	2,136 (1,998 to 2,277)
NHS costs, £: Model*	1,416 (1,056 to 1,887)	1,333 (993 to 1,780)	4,071 (2,600 to 6,379)
NHS costs, £: Lifetime*	3,552 (3,165 to 4,036)	5,298 (4,782 to 5,820)	8,036 (6,455 to 10,469)
Life expectancy, yrs	22.6 (22.0 to 23.2)	22.6 (22.0 to 23.2)	22.6 (22.0 to 23.2)
Years with early-stage DD	9.71 (6.38 to 12.05)	10.39 (7.23 to 12.72)	13.25 (10.52 to 15.61)
Number of courses of treatment for early-stage DD	0.0 (0.0 to 0.0)	1.0 (1.0 to 1.0)	3.65 (2.35 to 5.74)
Number of operations for late-stage DD	1.36 (1.0 to 1.88)	1.30 (0.94 to 1.79)	0.93 (0.63 to 1.32)
Incremental QALYs vs standard care*	N/A	0.048 (-0.053 to 0.166)	0.307 (0.110 to 0.524)†
Incremental cost vs standard care, £*	N/A	1,746 (1,418 to 2,060)†	4,484 (2,969 to 6,859)^†^
Cost/QALY vs standard care, £*	N/A	36,125 (dominated to 396,960)	14,593 (7,534 to 42,698)^†^

*Discounted at 3.5% per annum.

† p < 0.05 based on percentiles of probabilistic draws generated using the probabilistic sensitivity analysis described in Supplementary Methods 2.

DD, Dupuytren’s disease; N/A, not applicable; QALY, quality-adjusted life year.

One course of adalimumab increased mean QALYs by 0.048 (95% CI -0.053 to 0.166) and the mean cost was £1,746 higher (95% CI £1,418 to £2,060) than for standard care alone. Repeated courses of adalimumab gained a mean 0.307 QALYs (95% CI 0.110 to 0.524) and the mean cost was £4,484/patient (95% CI £2,969 to £6,859) higher than standard care. The incremental cost-effectiveness ratio for repeated courses of adalimumab compared with standard care was £14,593 (95% CI £7,534 to £42,698) per QALY gained. A combination of standard care and repeated courses of adalimumab showed extended dominance over one course of adalimumab, being less costly and generating more QALYs.

**Fig. 2:**
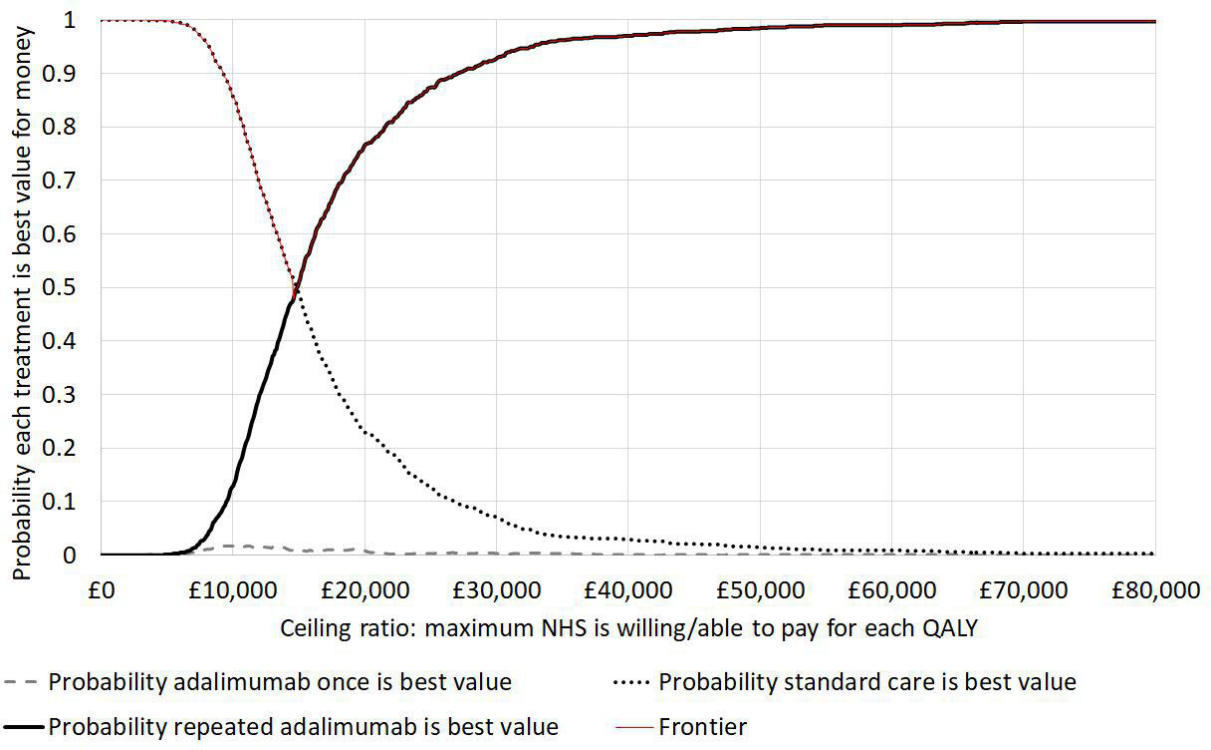
Cost-effectiveness acceptability curves showing the probability that each treatment strategy is best value for money at different values that the NHS may be willing or able to pay to gain one quality-adjusted life year (QALY). For example, if the NHS were willing to pay £30,000 per QALY gained, there is a 93% probability that repeated adalimumab is best value for money out of the three treatments shown, a 7% probability that standard care is best and < 1% probability that one course of adalimumab is best value for money. The frontier shows the probability of being cost-effective for the treatment that is expected to be best value for money at each ceiling ratio; the difference between the frontier and 1 indicates the decision uncertainty, i.e. the probability of adopting a treatment that is not in fact best value for money.

Probabilistic sensitivity analysis showed that if the NHS is willing to pay £20,000 per QALY gained,^14^ there is a 77% chance that repeated adalimumab courses are best value for money; this increased to 93% at a £30,000/QALY ceiling ratio (Figure 2, Supplementary Figures c and d). The expected value of perfect information that eliminates all uncertainty around the decision between the three treatment strategies was £292 per patient or £755 million for all 2,584,411 prevalent cases in the UK (Supplementary Table xii, Supplementary Figure e). This represents the maximum amount that it would be worth spending on future research in this area. The maximum value of a confirmatory trial that eliminated uncertainty around the parameters estimated from RIDD data, plus the duration of quiescence was £105 per patient or £272 million for the UK population.

One-way sensitivity analyses (Supplementary Figure b) demonstrated that changes in two model inputs could increase the cost per QALY gained for repeated adalimumab compared with standard care to > £20,000/QALY gained: reducing the utility gain associated quiescence to its lower 95% CI (0.0079), or reducing the duration of quiescence to 18 months. However, no single parameter change increased the cost-effectiveness ratio above £25,000/QALY and lower prices could reduce it.

We also compared adalimumab and standard care against a single course of radiotherapy and against steroid injections every six months in quiescent patients, using additional assumptions due to the limited data. This demonstrated that radiotherapy would be strongly dominated by adalimumab and have < 1% chance of being best value for money if it had the same efficacy as adalimumab, since it is more costly than one course of adalimumab (Supplementary Figures f and g, Supplementary Table xiii). At £341/course, giving three steroid injections to quiescent patients every six months would be less costly than giving adalimumab every three years if steroids were at least as effective.

## Discussion

There is currently no approved treatment for early-stage DD and patients are currently advised to wait until they have progressed and have flexion deformities limiting hand function before being offered surgery. The RIDD trial showed that a course of four adalimumab injections decreased nodule hardness and size.^9^ Here we assessed the cost-effectiveness of adalimumab for early-stage DD. Due to the low healthcare resource use, a difference in QALYs below the minimally important difference for EQ-5D^24^ and small numbers of patients progressing to surgery (ten in the standard care group, three in the adalimumab group),^9^ adalimumab was not cost-effective over a 12- to 18-month time horizon. However, DD is a slowly progressive disease and the RIDD trial found that nodules continued to soften and reduce in size nine months after the final injection.^9^ We would expect most of the health gains from treatment to be experienced more than 12 months after start of treatment, if those patients with reductions in nodule hardness and size are less likely to progress to late-stage DD and surgery. Therefore, we also performed long-term modelling.

The model showed that if adalimumab-induced quiescence lasted for three years, maintaining quiescence by further courses of four injections every three years would cost £14,593 per QALY gained compared with standard care. As treatments costing < £20,000/QALY are generally considered cost-effective,^14^ we can be 77% confident that repeated adalimumab is cost-effective compared with standard care (Figure 2). The cost-effectiveness ratio would fall to £8,508/QALY if the price was similar to etanercept (Supplementary Figure b). Adalimumab prices vary substantially between countries, up to $3,380 (£2,824) per dose in the USA,^25^ although this may change with the introduction of biosimilars in 2023. While the duration of quiescence is unknown, nodules continued to soften and shrink up to the 18-month trial follow-up;^9^ sensitivity analyses suggested that adalimumab would cost < £25,000/QALY gained even if quiescence lasted only 18 months.

RIDD is the largest randomized trial on early-stage DD^4^ and the first with prospective collection of costs and utilities. Key strengths of the within-trial economic evaluation include our comprehensive assessment of health and social services use and the robust estimation of unit costs. We achieved high levels of data completion, which reassures us that our results are both robust and representative of the trial population over the follow-up. Although there were more missing data at 18 months (partly attributable to the COVID-19 pandemic), we used multiple imputation to minimize the risk of bias. The COVID-19 pandemic may have affected quality of life and healthcare use after the 12-month follow-up. However, the observed data reflect the low healthcare use expected for early-stage DD, and we believe that the between-group comparisons presented are representative of our study population.

Only five previous model-based economic evaluations on DD have been published,^10,19^ and this is the first to model early-stage progressive DD. Our model allows for longer sequences of treatments than previous studies. We found that a strategy of up to three percutaneous needle fasciotomies, followed by limited fasciectomy, then dermofasciectomy was cost-effective (£1,435/QALY gained) compared with providing only best supportive care after percutaneous needle fasciotomy (Supplementary Table xiii).

RIDD evaluated surrogate outcome measures (nodule hardness and size) over 18 months. Increased nodule size has been shown to correlate with development of late-stage finger contractures,^17^ and nodule hardness has been used in other early-stage DD studies.^23,26,27^ While approximately ten years’ follow-up would be required to provide level 1 evidence linking quiescence at 18 months with development of flexion deformities, we observed higher EQ-5D utility in patients achieving quiescence (Table III) and more placebo-treated participants underwent/were awaiting surgery.^9^ Furthermore, when the nodules affected the proximal interphalangeal joint, placebo group participants developed greater flexion deformity.^9^ Taken together with evidence that anti-TNF downregulates myofibroblast activity in vitro^6,7^ and in vivo,^8^ our data suggest that intranodular injections of adalimumab are likely to control disease progression.^9^ Further follow-up would be needed to test the assumption that patients who are quiescent after the first course of treatment will remain quiescent following retreatment. Further research on the natural history of early-stage DD would also inform economic evaluations for other interventions. The analysis also focused on treatment and progression of one nodule, but in practice patients may have many nodules at different stages and surgery for late-stage DD may treat several nodules simultaneously. Although it relies on assumptions, our model indicates that adalimumab is likely to be cost-effective and highlights the areas where further research is likely to be most valuable. The introduction of an effective treatment for early-stage DD may increase referrals; this was not factored in the analysis since the magnitude is unknown.

There are no RCTs on intralesional steroid injections for early-stage DD and the only RCT on radiotherapy was unblinded and compared two radiotherapy doses.^5,9,28^ NICE recommend that radiotherapy should not be used for DD outside research settings.^22^ Our results comparing adalimumab against these treatments should therefore be interpreted with caution, and are intended only to give an indication of the spread of possible results and the value of further research.^11^ The analysis excluded complications from steroids and radiotherapy.^4,28^ However, our study suggests that there would be little value in further research comparing radiotherapy versus adalimumab.

Our results suggest that adalimumab is likely to be a cost-effective treatment for progressive early-stage DD and that additional research is likely to be good value for money. The value of eliminating all uncertainty around this decision was found to be £755 million in improvements in health of UK patients and NHS savings. Adalimumab therefore has the potential to transform the management of DD.

**Take home message**

- In the trial-based economic evaluation, adalimumab cost £503,410 per quality-adjusted life year (QALY) gained compared with standard care over 12 months.

- The model-based extrapolation showed that over a lifetime, repeated courses of adalimumab are likely to cost £14,593 per QALY gained.

- Repeated courses of adalimumab are likely to be a cost-effective way to treat early-stage Dupuytren’s disease, compared with standard care.
